# Differences in Lifestyle Behaviours of Students between Inner Urban and Peri-urban High Schools: A Cross-Sectional Study in Chongqing, China

**DOI:** 10.3390/ijerph17072282

**Published:** 2020-03-28

**Authors:** Zhengjie Cai, Ziwei Zhang, Mao Zeng, Jinli Xian, Xun Lei, Yong Zhao

**Affiliations:** 1 School of Public Health and Management, Chongqing Medical University, Chongqing 400016, China; 2018111015@stu.cqmu.edu.cn (Z.C.); zengmao@stu.cqmu.edu.cn (M.Z.); 2018111037@stu.cqmu.edu.cn (J.X.); leixun521@163.com (X.L.); 2 Research Center for Medicine and Social Development, Chongqing Medical University, Chongqing 400016, China; 3 The Innovation Center for Social Risk Governance in Health, Chongqing Medical University, Chongqing 400016, China; 4 The Second Clinical College, Chongqing Medical University, Chongqing 400016, China; 2017221022@stu.cqmu.edu.cn; 5 Chongqing Key Laboratory of Child Nutrition and Health, Chongqing 400016, China

**Keywords:** inner-urban, peri-urban, high school student, lifestyle behaviour

## Abstract

Background: Lifestyle behaviours of students from schools in different socioeconomic areas may be different. Few studies have investigated such topics in China. This study aimed to explore the differences in lifestyle behaviours between inner urban high school students (IUHSSs) and peri-urban high school students (PUHSSs). Methods: A cross-sectional survey based on a self-report questionnaire was administered among 1560 high school students (726 from inner urban high schools and 834 from peri-urban high schools) in Chongqing, China. Physical activity, sleep time, screen time and dietary behaviours were assessed according to a series of recommendations of Chinese guidelines. Results: No significant difference was found in meeting the recommendation for daily physical activity between IUHSSs and PUHSSs (7.6% vs. 6.8%, *p* > 0.05). PUHSSs were more likely to meet the recommendations of weekdays’ sleep time (14.9% vs. 5.4%, *p* < 0.001), weekdays’ and weekends’ screen time (85.4% vs. 76.7%, *p* < 0.001; 21.1% vs. 14.3%, *p* < 0.001), and had higher proportion of high-score group of dietary behaviours (58.6% vs. 36.4%, *p* < 0.001) than those of IUHSSs. IUHSSs were more likely to meet the recommendation of weekends’ sleep time (75.6% vs. 67.9%, *p* < 0.001) than that of PUHSSs. Conclusions: A low proportion met the recommendations of physical activity, weekdays’ sleep time and weekends’ screen time among high school students in Chongqing, China. Lifestyle behaviours may differ between inner urban and peri-urban high school students. Additional support or targeted health education should be provided by high schools to improve the lifestyle behaviours of students, especially in inner urban districts.

## 1. Introduction

In recent years, increasing evidence has indicated that the prevalence of overweight and obesity among children and adolescents is high and has been continuously rising [1], especially in China [2]. Healthy lifestyle behaviours are considered as essential roles in the prevention and control of obesity and health promotion [3]. On this basis, healthy lifestyle behaviours of children and adolescents must be considered and promoted by public health decision-makers, school principals and management bodies.

Various unhealthy lifestyle behaviours, such as a high level of sedentary behaviour, low level of physical activity and poor dietary behaviours, frequently emerge during the period of adolescence and young adulthood [4]. The World Health Organisation estimated that globally, more than 80% of adolescents had insufficient physical activity [5], and 80–89% of Chinese adolescents did not meet the current recommendation of physical activity guidelines [6]. Regarding sleep and screen time, previous studies have shown that adolescents may be vulnerable to short sleep duration [7], which has been associated with physical health problems, such as obesity, insulin resistance, and a higher risk for cardiovascular abnormalities [8,9,10]. More than 60% of Chinese adolescents aged 6 to 17 years had less than 8 h of sleep, according to the Chinese Sleep Research Society [11]. With the rapid development of the Internet and smartphones, adolescents have considerable on-screen exposure [12]. Moreover, adolescents are not following the recommended dietary practices, such as ‘drinking milk or dairy products every day’ and ‘eating vegetables every day’ [13], and they are undergoing a rapid nutrition transition to a high-fat diet [14].

In the context of metropolitan China, ‘urban’ refers to inner urban and peri-urban areas. Peri-urbanisation refers to the dispersal of urban growth towards the rural surroundings (urban sprawl), thereby creating landscapes that are characterised by combined social and economic activities of urban and rural areas [15]. Chongqing is a municipality in mid-western China, including 9 inner urban districts and 17 peri-urban districts. To date, individual-level indicators have been mainly used to explore the associations between socioeconomic status and adolescents’ health behaviours. Some studies have demonstrated that adolescents living in rural areas had a higher level of physical activity, shorter sitting time, less exposure to computers, video games and television, more calorie intake and less fruit and vegetable consumption compared with those living in the urban areas [16,17,18]. Limited studies have investigated the differences in students’ lifestyle behaviours between schools in different socioeconomic districts [19,20]. Moreover, previous studies have explored the lifestyle behaviours of children and adolescents [21,22]. However, few studies focused on multiple outcome indicators of lifestyle behaviours of high school students.

With the social, cultural, and educational context of China, the heavy burden of study could make it more difficult for Chinese high school students to maintain healthy behaviours over the course of their three-year program [23]. Hence, this study aimed to (1) assess the lifestyle behaviours (daily physical activity, weekdays’ sleep time, weekends’ sleep time, weekdays’ screen time, weekends’ screen time and dietary behaviours) of high school students; (2) explore the differences in lifestyle behaviours between inner urban high school students (IUHSSs) and peri-urban high school students (PUHSSs). The results of this study could provide insights into the unhealthy lifestyle behaviours of high school students and the associated factors, and serve as a reference for conducting targeted health education of this population.

## 2. Materials and Methods

### 2.1. Study Design and Sample Size

A cross-sectional survey of lifestyle behaviours was administered in June 2019 among high school students from one inner urban and two peri-urban high schools in Chongqing.

Literature demonstrated that the incidence of unhealthy lifestyle behaviours among middle school students in Nanjing, China, was approximately 19.9% [24]. According to the formula of sample size calculation

(1) $$N=(Z_{\alpha }{}^{2}\times p\times q)/d^{2}$$

we set *p* = 0.199, *q* = 1 − *p* = 0.801, and margin of error *d* = 0.10 × *p* = 0.0199, *Z_a_* = 1.96; the calculated sample size was 1530. In the survey, the actual total sample size in the survey included 1560 individuals.

### 2.2. Participants

Four-stage stratified cluster random sampling was used to recruit participants. High school students in grades 10 and 11 were eligible to participate in this study. First, one district from 9 inner urban districts and two districts from 17 peri-urban districts were selected. Second, one high school was selected from each designated district. Third, 6–12 classes were selected from each high school, with approximately 60 students in each class. Finally, all students from selected classes in these schools were invited to participate in this study. The exclusion criteria were students who had a history of major diseases, chronic health condition, or mental trauma. Investigators briefly explained the research and distributed the questionnaire to all students. The students were asked to fill out the anonymous questionnaire within approximately 15–20 min before or after a lecture (as approved by their class teacher), and each student was instructed to sign an informed consent before the questionnaire survey. A total of 1722 students participated in this study, and 1658 responses were obtained with a response rate of 96.3%. Owing to the incomplete answers to the questionnaire (*n* = 98), 1560 high school students were included in the analysis. And written informed consent for processing personal data was obtained from each participant. This study was approved by the Ethics Committee of Chongqing Medical University (record number 2016001).

### 2.3. Survey Questionnaire

#### 2.3.1. Demographic Characteristics

Data on demographic characteristics included age, gender (male/female), residence (city/rural), lack of siblings (yes/no) and boarding school (yes/no). Average monthly living expenses (RMB) were divided into three categories (low: ≤800; medium: 801–1200; and high: >1200) [25]. Parents’ educational level was divided into three categories (low: primary school or below; medium: secondary school/secondary vocational school/high school; high: college or above) [26]. Two questions about the grandparents’ co-residence and primary dietary caregivers of the students were designed, which were ”how many grandparents live with you?”, response options were ‘0′, ‘1′, and ‘2/more’ and “who is in charge of your diet (except for school meals)?”, response options were ‘parents’, ‘grandparents’, and ‘others’.

#### 2.3.2. Lifestyle Behaviours

Lifestyle behaviours data included physical activity, sleep time, screen time and dietary behaviours (eating habits and beverage consumption). A set of questions on physical activity, sleep and screen time were adapted from the China Health and Nutrition Survey [27], and questions on dietary behaviours were adapted from the Chinese Dietary Guideline 2016 and Dietary Guidelines for Chinese School-Age Children 2016 [28,29]. These questions were set as follows: 1) Do you participate in any relatively intense physical exercises, such as volleyball, soccer and badminton? The response options were ‘yes’ or ‘no’. If the response was ‘yes’, then we would ask how many times do you participate in these physical exercises each week? And on average, how long do you participate in these physical exercises each time? (hours: minutes). 2) How many hours each day do you usually sleep on weekdays, including daytime and night time? (hours). 3) How many hours each day do you usually sleep on weekends, including daytime and night time? (hours). 4) On average, how long is your daily screen time on weekdays, such as watching TV, online videos, using a computer or smartphone and playing video games? (hours: minutes). 5) On average, how long is your daily screen time on weekends? (hours: minutes). 6) Do you pay attention to having a light diet (the limit of salt and cooking oil intake)? 7) Do you wash hands before eating? 8) Do you watch TV while eating? 9) Do you eat fried foods? 10) Do you eat excessively during a meal? 11) Do you eat eggs without egg yolk? 12) Do you eat smoked food? 13) Do you eat sweet food? The response for questions 6–13 had five options (always, usually, sometimes, occasionally, seldom). 14) How often do you drink carbonated drinks? 15) How often do you drink fruit and vegetable juice drinks (not pure juice drinks)? 16) How often do you drink sports drinks? 17) How often do you drink tea beverage? 18) How often do you drink milk beverage? 19) How often do you drink vegetable protein drinks? The response options for questions 15–19 were ‘always (>5 times/week)’, ‘usually (3–4 times/week)’, ‘sometimes (1–2 times/week)’, ‘occasionally (1–3 times/month)’, ‘seldom (<once/month)’.

### 2.4. Outcome Assessment

#### 2.4.1. Physical Activity

As is stated by the physical activity guideline for Chinese children and adolescents [30], children and adolescents aged 6–17 years should have at least 1 h moderate-to-vigorous physical activity per day. Average daily physical activity was categorised into <1 h and ≥1 h in this study.

#### 2.4.2. Sleep Time

As recommended by the Chinese dietary guideline 2016 [28] and health requirements of daily learning time for secondary and elementary school students [31], high school students should get at least 8 h of sleep a day. Sleep durations on weekdays and weekends were categorised into ≥8 h and <8 h in this study.

#### 2.4.3. Screen Time

In accordance with the physical activity guideline for Chinese children and adolescents [30], daily screen time of high school students should be less than 2 h. Screen time on weekdays and weekends were categorised into <2 h and ≥2 h in this study.

#### 2.4.4. Dietary Behaviours

For the items of eating habits, the five response options for questions 6 and 7 (seldom, occasionally, sometimes, usually, always) scored ranging from 1 to 5, respectively, and the five response options for questions 8–13 (always, usually, sometimes, occasionally, seldom) scored ranging from 1 to 5, respectively. Regarding beverage consumption, the five response options for questions 14–19 ‘always (>5 times/week)’, ‘usually (3–4 times/week)’, ‘sometimes (1–2 times/week)’, ‘occasionally (1–3 times/month)’, ‘seldom (<once/month)’ scored ranging from 1 to 5, respectively. The score of dietary behaviours was constructed based on the sum of the recorded responses for these items, and the total score ranged from 14 to 70. According to the frequency distribution of the dietary behaviours’ score, students with a score less than 52 (51.7%) were defined as the low-score group, and students with a score equal or higher than 52 (48.3%) were defined as the high-score group.

### 2.5. Quality Control

All investigators in this study were recruited to join the investigation team via rigorous interview. They were uniformly trained and required to have a good understanding of the approach, objectives and methodology of this study, and had extensive experience in dealing with potentially sensitive issues. Many investigators were arranged on the spot to explain the questions in the questionnaire that the students did not understand. Moreover, team members communicated with teachers in advance to obtain their support and understanding. The collected questionnaires were reviewed by investigators to ensure efficiency. The data were double-entered in EpiData 3.1 software (Jens M. Lauritsen, Odense, Denmark).

### 2.6. Statistical Analyses

All statistical analyses were performed using STATA software (Version 12, StataCorp, College Station, TX, USA). Invalid or missing data were excluded, and all data were double-checked. Owing to the non-normal distribution of age, it was described using median and inter-quartile range (IQR), and the other demographic characteristics were categorical variables, which were described using frequency and percentiles. The Wilcoxon rank sum test was conducted to examine the age difference between IUHSSs and PUHSSs, and χ^2^ tests were conducted to examine the differences between IUHSSs and PUHSSs in terms of other demographic characteristics and four lifestyle behaviours (physical activity, sleep time, screen time and dietary behaviours’ score). All outcome indicators were transformed into binary variables. A forward stepwise logistic regression analysis was performed to assess the factors influencing the four lifestyle behaviours of high school students. Odds ratios (OR) and their corresponding 95% confidence intervals (CI) were reported. *p* < 0.05 was considered statistically significant (two-sided).

## 3. Results

### 3.1. Demographic Characteristics of Participants

The demographic characteristics of the participants are provided in Table 1. The total of 1560 high school students included 726 IUHSSs and 834 PUHSSs. The average age was 16.3 years. The results showed that all demographic characteristics varied between IUHSSs and PUHSSs except for gender (*p* < 0.001).

### 3.2. Comparison of Lifestyle Behaviours between IUHSSs and PUHSSs

Table 2 shows the comparison of lifestyle behaviours between IUHSSs and PUHSSs. Only 7.6% of IUHSSs and 6.8% of PUHSSs met the recommendation for daily physical activity. Only 5.4% of IUHSSs and 14.9% of PUHSSs had ≥8 h/d sleep time on weekdays. Only 14.3% of IUHSSs and 21.1% of PUHSSs had <2 h/d screen time on weekends. And 36.4% of IUHSSs and 58.6% of PUHSSs obtained a high score for dietary behaviours. Statistically significant differences were observed in lifestyle behaviours between IUHSSs and PUHSSs except for average daily physical activity (*p* < 0.05). Supplementary Table S1 presents a comparison of each item of dietary behaviours between IUHSSs and PUHSSs. Among the six types of beverage consumption, students who always or usually (≥3 times/week) drank carbonated drinks, fruit and vegetable juice drinks (not 100% juices drinks), sports drinks, tea beverage, milk beverage and vegetable protein drinks accounted for 9.3%, 16.0%, 6.0%, 12.0%, 23.3% and 20.1%, respectively. Statistically significant differences were observed in each item of dietary behaviours between IUHSSs and PUHSSs except paying attention to having a light diet (the limit of salt and cooking oil intake), eating egg without yolk and drinking milk beverage (*p* < 0.05).

### 3.3. Stepwise Logistic Regression for Identifying Factors Affecting the Lifestyle Behaviours of High School Students

**Figure 1:**
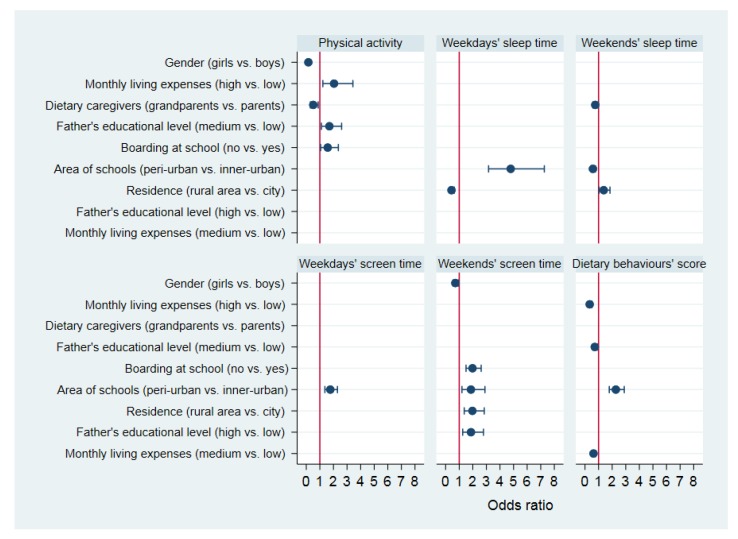
Stepwise logistic regression analysis for identifying factors affecting the lifestyle behaviours of high school students (*n* = 1560).

The results of stepwise logistic regression analysis are shown in Figure 1. We found that PUHSSs were more likely to meet the recommendations of weekdays’ sleep time (OR: 4.79; 95% CI: 3.16, 7.26), weekdays’ screen time (OR: 1.77; 95% CI: 1.37, 2.29), weekends’ screen time (OR: 1.86; 95% CI: 1.20, 2.89), and had a higher proportion of high score of dietary behaviours (OR: 2.27; 95% CI: 1.79, 2.89) than those of IUHSSs. While IUHSSs were more likely to meet the recommendation of weekends’ sleep time (OR: 0.58; 95% CI: 0.44, 0.77) than that of PUHSSs. In addition, gender, average monthly living expenses, primary dietary caregivers (except for school meals), fathers’ educational level, boarding at school and residence were associated with the lifestyle behaviours of high school students. Girls were less likely to adhere to the recommendations of physical activity (OR: 0.15; 95% CI: 0.09, 0.27) and weekends’ screen time (OR: 0.72; 95% CI: 0.55, 0.94) than boys. Students with high monthly living expenses had a higher adherence of physical activity guideline (OR: 2.04; 95% CI: 1.22, 3.43) and students with high or medium monthly living expenses had a lower proportion of high score of dietary behaviours (OR: 0.35; 95% CI: 0.23, 0.51 and OR: 0.63; 95% CI: 0.50, 0.79) than those with low monthly living expenses. Students, whose grandparents were primary dietary caregivers, were less likely to adhere to the recommendations of daily physical activity (OR: 0.51; 95% CI: 0.30, 0.88) and weekends’ sleep time (OR: 0.76; 95% CI: 0.59, 0.97) compared with those whose parents were dietary primary caregivers. Students, whose father had a medium education level, were more likely to meet the recommendation of daily physical activity (OR: 1.70; 95% CI: 1.11, 2.60), and had a lower proportion of high score of dietary behaviours (OR: 0.72; 95% CI: 0.57, 0.91) than those whose father had a low education level. Students whose father had high education level were more likely to meet the recommendation of weekends’ screen time (OR: 1.86; 95% CI: 1.25, 2.78) than those whose father with low education level. Boarding school students were more likely to meet the recommendations of daily physical activity (OR: 1.58; 95% CI: 1.05, 2.36) and weekends’ screen time (OR: 1.98; 95% CI: 1.50, 2.61) than those who lived at home. Students who lived in rural areas were more likely to meet the recommendations of weekends’ sleep time (OR: 1.38; 95% CI: 1.04, 1.84) and weekends’ screen time (OR: 1.97; 95% CI: 1.37, 2.84), and were less likely to adhere to the recommendation of weekdays’ sleep time (OR: 0.43; 95% CI: 0.30, 0.63) than those living in urban areas. The Hosmer–Slideshow goodness-of-fit test indicated that all the logistic regression models had good fits (*p* > 0.05) except for the logistic regression model for identifying factors affecting the weekends’ sleep time of high school students. And the Nagelkerke R^2^ value of the six stepwise logistic regression models were 0.15, 0.10, 0.03, 0.04, 0.08, 0.10, respectively.

## 4. Discussion

Our results showed that a low proportion met the recommendations of physical activity, weekdays’ sleep time and weekends’ screen time among high school students in Chongqing, China. And remarkably, differences were found in lifestyle behaviours between IUHSSs and PUHSSs. The findings in this study revealed that efforts are needed to conduct targeted health education by high schools to improve the lifestyle behaviours of students, especially in inner urban districts.

The present study showed that only 7.6% of IUHSSs and 6.8% of PUHSSs met the recommendation of physical activity, which was similar to the physical activity level of adolescents aged 15–19 in Shanghai, China (5.7%) [32]. In China, exam-oriented education is common, and schools give top priority to academic achievements. Many schools have reduced the physical education curriculum to maximise instructional time for the main examination subjects (such as mathematics, English) and occupied the recess and other physical activity breaks for extracurricular study [33,34]. This phenomenon is particularly prominent in high schools, where high school students need to prepare for the National College Entrance Examination.

The results showed that 5.4% of IUHSSs and 14.9% of PUHSSs had sleep time ≥8 h/d on weekdays. A previous study demonstrated that the average bedtime, getting up time in the morning and sleep duration were approximately 10:30 pm, 5:45 am and 7 h, respectively, among high school students in China [35]. High school students go to bed late and get up early to meet school schedules, although their sleep duration is not enough for physiological requirements [36], resulting in severe sleep debt and daytime sleepiness. Furthermore, we found a higher proportion of ≥8 h/d sleep time on weekends than on weekdays, IUHSSs and PUHSSs accounted for 75.6% and 67.9%, respectively. This phenomenon was similar to medical university students with heavy academic pressure [37]. The results demonstrated that the actual sleep requirement of high school students was not enough on weekdays, or this could be an indicator of ‘catch-up’ sleep to compensate for sleep insufficiency on weekdays. On the contrary, a lower proportion of <2 h/d screen time on weekends than on weekdays was observed in this study, with IUHSSs and PUHSSs accounting for 76.7% and 85.4% on weekdays, 14.3% and 21.1% on weekends, respectively. Excessive screen time of high school students on weekends may have been ignored by previous studies [33,34]. And similar to sleep time, screen time seems to have a rebound among high school students on weekends. This condition occurs possibly because high school students are not allowed to use mobile phones or go to an Internet cafe on weekdays, while these situations could happen due to the break time on weekends.

Regarding the results of dietary behaviours, a noteworthy finding was that milk beverage consumption of ≥ 3 times/week obtained the highest proportion among the six surveyed beverages. Milk beverage cannot be equivalent to milk or dairy products. The main ingredient of most milk beverage is water, and their nutritional value is lower than milk or dairy products [29]. A previous study demonstrated that most parents held the misconception that milk beverage had the same nutritional value as milk for children and adolescents and can be used as long-term substitutes for milk [38], thereby resulting in the high consumption of milk beverage. Moreover, the consumption proportions of ≥3 times/week among the four surveyed sugar-sweetened beverages (SSBs) was consistent with results of one previous study, which showed that vegetable/fruit-flavoured drinks and tea beverage had become the leading SSBs in China [39]. With increasing studies and rapid spread of mass media, the public has improved the awareness of the high sugar content and hazards of carbonated SSBs (CSSBs) [40,41]. Thus, some people prefer non-carbonated SSBs (NCSSBs) as potential alternatives to CSSBs with the perception that they are healthier [42]. However, high sugar and energy contents in NCSSBs categories were still found [43].

Prevention and lifestyle interventions for adolescents’ obesity were primarily conducted through schools to reach a wide population, but the outcomes of many school-based interventions were equivocal [44]. A previous study reported that students from high schools with a high school-level socioeconomic status were more likely to eat breakfast and less likely to drink SSBs [20]. In contrast to the results in western countries, PUHSSs were more likely to have better lifestyle behaviours than IUHSSs in this study. We have concluded the following findings and reasons. First, the effectiveness of health education implementation may vary at different socioeconomic school levels. Owing to regional and economic development, outstanding teachers in inner urban schools accounted approximately for two-thirds in Chongqing [45], and high schools in inner urban may focus more on academic performance for school reputation compared with peri-urban high schools, but ignore the health lifestyle behaviours of students. IUHSSs may have more pressure and study longer time per day than PUHSSs. This condition can explain why PUHSSs were more likely to meet the recommendations of weekdays’ sleep time than that of IUHSSs. Second, availability, affordability and accessibility of food and screen devices could explain the differences in screen time and dietary behaviours between IUHSSs and PUHSSs. PUHSSs were more likely to meet the recommendations of screen time on weekdays and weekends, possibly because students from more urbanised school districts had greater access to screen devices and would be more likely to engage in screen time behaviours. And PUHSSs were more likely to have a higher proportion of high-score of dietary behaviours than that of IUHSSs. The reason could be that the increasing access to food stores and fast-food restaurants in inner urban areas, resulting in excessive intake of fried food, sweet food and SSBs. Third, peer communication gradually replaced parent–child communication as a key influencing factor of adolescent behaviours [46], which may also be related to adolescents’ lifestyle behaviours. High school students who attend inner-urban schools might be exposed to peers who sacrifice sleep to study or peers who consume many snack foods and beverages. A noteworthy finding was that IUHSSs were more likely to obtain ≥8 h/d sleep on weekends than that of PUHSSs. One possible explanation was that most PUHSSs boarded at school, whereas most IUHSSs lived at home according to the results in this study. On weekends, the rules and regulations of schools made boarding school students get up and go to bed on time, whereas students who lived at home could oversleep. In terms of physical activity, no remarkable difference was found between IUHSSs and PUHSSs. Combined with the extremely low level of physical activity of high school students in this study, this might reflect, at least partly, that the physical education curriculum of high school students was neglected both in inner urban and peri-urban schools. Hence, additional support or targeted health education for the benefit of sufficient sleep, appropriate screen time, positive leisure activities and healthy dietary behaviours should be provided by schools to raise awareness among students, parents and teachers, especially in inner urban high schools.

Gender, average monthly living expenses, primary dietary caregivers, boarding at school, fathers’ educational level and residence were correlated with lifestyle behaviours of high school students. Boys were more likely to adhere to the recommendations of physical activity and weekends’ screen time than girls, which is in line with previous studies [47,48]. This could be partially explained by different perceived social gender roles for boys and girls. Boys appear to receive more family encouragement and support to be physically active than girls [49], whereas girls are more likely to stay at home [50]. Students with high monthly living expenses had a higher level of physical activity and a lower proportion of high-score of dietary behaviours than those with low monthly living expenses. Monthly living expenses may affect the affordability of sports equipment and food to a certain degree, and students with high living expenses may be more likely to purchase sports equipment, thereby increasing the opportunity and feasibility of participating in physical activities. Simultaneously, the opportunity to purchase food, such as snacks and SSBs, was likely to increase. In addition, students whose parents were primary dietary caregivers and boarding school students were more likely to adhere to the recommendation of daily physical activity compared with those whose grandparents were dietary primary caregivers and the students who lived at home. Parental and peer social support was associated with healthy physical activity behaviours in adolescents [51]. Previous studies reported that a strong link was found between parental support and the level of children’s physical activity, and physical activity in parents was associated with greater physical activity in children [52]. However, growing evidence indicated that grandparents were the primary caregivers of children and adolescents [53,54]. Grandparents may avoid or not allow students to exercise actively for fear of him/her getting hurt. Moreover, there may be little chance that they could play sports with students because of their age. A previous study showed that peers had a remarkable effect on the levels of physical activity between each other [55]; several boarding school students lived in the same dormitory, and peer support may be more pronounced than students who lived at home. Moreover, parents as primary dietary caregivers and boarding school students were more likely to adhere to the recommendation of screen time on weekends. One possible explanation was that stricter rules of screen time were set for these students compared with those students whose grandparents as their primary caregivers and lived at home.

This study had certain limitations. First, the use of cross-sectional survey data reduced the researchers’ ability to make direct causal inferences. Future longitudinal studies should be conducted to confirm the findings. Second, the fact that students were from different grades in the same high school was not considered. Only grade one in inner urban high school and grade two in peri-urban high school were selected in this study. Future research needs to design and make the study sampling structure more reasonable. Third, the study relied on self-report, which may introduce biases caused by dishonesty and measurement flaws. Fourth, this questionnaire was designed with reference to the questionnaire of the China Health and Nutrition Survey, Chinese Dietary Guideline 2016 and the Dietary Guidelines for Chinese School-Age Children 2016. Still, the use of unvalidated questionnaires of the four outcomes were a limitation in this study, and we will strengthen the design and verification of the questionnaire in future study. The items of dietary behaviours were designed based on the Chinese dietary guidelines, which may not be representative in other countries and districts, further global collaborative researches on health behaviours are warranted. Fifth, we mainly considered the difference of screen-time based sedentary behaviour between IUHSSs and PUHSSs. However, sedentary behavior is the independent health risk, non-screen sedentary time of high school students, such as daily studying time, are also important and need to be explored in further study. Finally, the low percentage of explained variance of the stepwise logistic regression models was a limitation, especially the stepwise logistic regression models for identifying factors affecting the weekends’ sleep time and weekdays’ screen time of high school students. Other important factors influencing the healthy behaviours of high school students should be identified, and sample size should be expanded in further study.

## 5. Conclusions

A low proportion of meeting the recommendations of physical activity, weekdays’ sleep time and weekends’ screen time was observed among high school students in Chongqing, China. Compared with IUHSSs, PUHSSs were more likely to meet the recommendations of weekdays’ sleep time, weekdays’ and weekends’ screen time and had higher scores of dietary behaviours. Additional support or targeted health education for sufficient sleep, appropriate screen time, positive leisure activities and healthy dietary behaviours should be provided by high schools to raise the awareness of a healthy lifestyle among students, parents and teachers, especially in inner urban districts.

## Figures and Tables

**Table 1:** Demographic characteristics between inner urban high school students (IUHSSs) and peri-urban high school students (PUHSSs) in Chongqing, China (*n* = 1560).

Variables	Total (*n* = 1560) *n* (%)	Inner Urban (*n* = 726) *n* (%)	Peri-Urban (*n* = 834) *n* (%)	*Z/χ* ^2^	Contingency Coefficient	*p*
Age (Median (IQR))	16.0 (1.0)	16.0 (1,0)	17.0 (0.0)	−30.762	/	<0.001
Gender						
Boys	781 (50.1)	373 (51.4)	408 (48.9)	0.94	0.02	0.333
Girls	779 (49.9)	353 (48.6)	426 (51.1)			
Residence						
City	1,019 (65.3)	703 (96.8)	316 (37.9)	595.26	0.53	<0.001
Rural	541 (34.7)	23 (3.2)	518 (62.1)			
Lack of siblings						
Yes	553 (35.5)	436 (60.1)	117 (14.0)	359.32	0.43	<0.001
No	1007 (64.5)	290 (39.9)	717 (86.0)			
Boarding at school						
Yes	902 (57.8)	320 (44.1)	582 (69.8)	105.17	0.25	<0.001
No	658 (42.2)	406 (55.9)	252 (30.2)			
Average monthly living expenses (RMB)						
Low	911 (58.4)	307 (42.3)	604 (72.4)	207.03	0.34	<0.001
Medium	480 (30.8)	266 (36.6)	214 (25.7)			
High	169 (10.8)	153 (21.1)	16 (1.9)			
Father’s educational level						
Low	168(10.8)	14 (1.9)	154 (18.5)	605.57	0.53	<0.001
Medium	886(56.8)	254 (35.0)	632 (75.8)			
High	56 (32.4)	458 (63.1)	48 (5.8)			
Mother’s educational level						
Low	297 (19.0)	21 (2.9)	276 (33.1)	560.09	0.51	<0.001
Medium	848 (54.4)	322 (44.4)	526 (63.1)			
High	415 (26.6)	383 (52.8)	32 (3.8)			
Grandparents’ co-residence in the household						
0	874 (56.0)	461 (63.5)	413 (49.5)	30.78	0.14	<0.001
1	296 (19.0)	114 (15.7)	182 (21.8)			
2/more	390 (25.0)	151 (20.8)	239 (28.7)			
Main dietary caregivers (except for school meals)						
Mother/father	882 (56.5)	440 (60.6)	442 (53.0)	32.55	0.14	<0.001
Grandmother/grandfather	414 (26.5)	144 (19.8)	270 (32.4)			
Others	264 (16.9)	142 (19.6)	122 (14.6)			

**Table 2:** The comparison of lifestyle behaviours between IUHSSs and PUHSSs in Chongqing, China (*n* = 1560).

Variables	Total (*n* = 1560) *n* (%)	Inner Urban (*n* = 726) *n* (%)	Peri-Urban (*n* = 834) *n* (%)	χ^2^	Contingency Coefficient	*P*
Average daily physical activity						
<1 h	1448 (92.8)	671 (92.4)	777 (93.2)	0.32	0.01	0.572
≥1 h	112 (7.2)	55 (7.6)	57 (6.8)			
Weekdays’ sleep time						
≥8 h	163 (10.5)	39 (5.4)	124 (14.9)	37.41	0.15	<0.001
<8 h	1397 (89.6)	687 (94.6)	710 (85.1)			
Weekends’ sleep time						
≥8 h	1115 (71.5)	549 (75.6)	566 (67.9)	11.45	0.09	0.001
<8 h	445 (28.5)	177 (24.4)	268 (32.1)			
Weekdays’ screen time						
<2 h	1269 (81.4)	557 (76.7)	712 (85.4)	19.14	0.11	<0.001
≥2 h	291 (18.6)	169 (23.3)	122 (14.6)			
Weekends’ screen time						
<2 h	280 (18.0)	104 (14.3)	176 (21.1)	12.11	0.09	0.001
≥2 h	1280 (82.0)	622 (85.7)	658 (78.9)			
Dietary behaviours’ score						
High-score group	753 (48.3)	264 (36.4)	489 (58.6)	77.09	0.22	<0.001
Low-score group	807 (51.7)	462 (63.6)	345 (41.4)			

## Supplementary Materials

The following are available online at https://www.mdpi.com/1660-4601/17/7/2282/s1, Table S1: The comparison of dietary behaviours between IUHSSs and PUHSSs in Chongqing, China (*n* = 1560).
