# Supplementary material for: Differences in Lifestyle Behaviours of Students between Inner Urban and Peri-urban High Schools: A Cross-Sectional Study in Chongqing, China
Source: Int J Environ Res Public Health. 2020 Mar 28;17(7):2282. doi: 10.3390/ijerph17072282 (PMC7177544; doi:10.3390/ijerph17072282)
Supplement: Supplementary file 1 [file ijerph-17-02282-s001.pdf]

International Journal of
Environmental Research and Public Health

MDPI

Table S1 The comparison of dietary behaviours between inner urban high school students (IUHSSs) and peri-urban high school students (PUHSSs) in Chongqing, China (*n* = 1560).

Variables	Total (*n* = 1560) *n* (%)	Inner-urban (*n* = 726) *n* (%)	Peri-urban (*n* = 834) *n* (%)	χ^2	Contingency coefficient	*p*
Paying attention to having a light diet (the limit of salt and cooking oil intake)						
Always	144 (9.2)	74 (10.2)	70 (8.4)	9.23	0.08	0.056
Usually	271 (17.4)	124 (17.1)	147 (17.6)			
Sometimes	636 (40.8)	270 (37.2)	366 (43.9)			
Occasionally	346 (22.2)	176 (24.2)	170 (20.4)			
Seldom	163 (10.4)	82 (11.3)	81 (9.7)			
Watching TV while eating						
Always	78 (5.0)	47 (6.5)	31 (3.7)	27.35	0.13	<0.001
Usually	172 (11.0)	97 (13.4)	75 (9.0)			
Sometimes	390 (25.0)	192 (26.4)	198 (23.7)			
Occasionally	542 (34.7)	249 (34.3)	293 (35.1)			
Seldom	378 (24.2)	141 (19.4)	237 (28.4)			
Washing hands before eating						
Always	455 (29.2)	189 (26.0)	266 (31.9)	19.41	0.11	0.001
Usually	396 (25.4)	176 (24.2)	220 (26.4)			
Sometimes	413 (26.5)	191 (26.3)	222 (26.6)			
Occasionally	245 (15.7)	141 (19.4)	104 (12.5)			
Seldom	51 (3.3)	29 (4.0)	22 (2.6)			
Eating fried foods						
Always	46 (3.0)	25 (3.4)	21 (2.5)	21.61	0.24	<0.001
Usually	227 (14.5)	135 (18.6)	92 (11.0)			
Sometimes	717 (46.0)	316 (43.5)	401 (48.1)			
Occasionally	526 (33.7)	235 (32.4)	291 (34.9)			
Seldom	44 (2.8)	15 (2.1)	29 (3.5)			
Eating too much at a meal						
Always	96 (6.2)	51 (7.0)	45 (5.4)	16.19	0.10	0.006
Usually	238 (15.3)	129 (17.8)	109 (13.1)			
Sometimes	572 (36.7)	265 (36.5)	307 (36.8)			
Occasionally	525 (33.7)	237 (32.6)	288 (34.5)			
Seldom	129 (8.2)	44 (6.1)	85 (10.2)			
Eating egg without yolk						
Always	112 (7.2)	58 (8.0)	54 (6.5)	6.69	0.07	0.153
Usually	71 (4.6)	36 (5.0)	35 (4.2)			
Sometimes	136 (8.7)	73 (10.1)	63 (7.6)			
Occasionally	233 (14.9)	112 (15.4)	121 (14.5)			
Seldom	1008 (64.6)	447 (61.6)	561 (67.3)			
Eating smoked food						
Always	32 (2.1)	17 (2.3)	15 (1.8)	30.79	0.14	<0.001
Usually	123 (7.9)	64 (8.8)	59 (7.1)			
Sometimes	611 (39.2)	231 (31.8)	380 (45.6)			
Occasionally	624 (40.0)	325 (44.8)	299 (35.9)			
Seldom	170(10.9)	89 (12.3)	81 (9.7)			
Eating sweet food						
Always	131 (8.4)	78 (10.7)	53 (6.4)	61.06	0.19	<0.001
Usually	400 (25.6)	241 (33.2)	159 (19.1)			
Sometimes	591 (37.9)	243 (33.5)	348 (41.7)			
Occasionally	404 (25.9)	153 (21.1)	251 (30.1)			
Seldom	34 (2.2)	11 (1.5)	23 (2.8)			
Drinking carbonated drinks						
Always (>5 times/week)	47 (3.0)	29 (4.0)	18 (2.2)	40.20	0.16	<0.001
Usually (3–4 times/week)	99 (6.3)	62 (8.5)	37 (4.4)			
Sometimes (1–2 times/week)	263 (16.9)	150 (20.7)	113 (13.5)			
Occasionally (1–3 times/month)	518 (33.2)	237 (32.6)	281 (33.7)			
Seldom (<once/month)	633 (40.6)	248 (34.2)	385 (46.2)			
Drinking fruit and vegetable juice drinks (not 100% juices drinks)						

Always (>5 times/week)	90 (5.8)	57 (7.9)	33 (4.0)	96.99	0.24	<0.001
Usually (3–4 times/week)	144 (9.2)	93 (12.8)	51 (6.1)			
Sometimes (1–2 times/week)	340 (21.8)	200 (27.5)	140 (16.8)			
Occasionally (1–3 times/month)	471 (30.2)	214 (29.5)	257 (30.8)			
Seldom (< once/month)	515 (33.0)	162 (22.3)	353 (42.3)			
Drinking sports drinks						
Always (>5 times/week)	27 (1.7)	16 (2.2)	11 (1.3)	60.08	0.19	<0.001
Usually (3–4 times/week)	67 (4.3)	50 (6.9)	17 (2.0)			
Sometimes (1–2 times/week)	142 (9.1)	90 (12.4)	52 (6.2)			
Occasionally (1–3 times/month)	300 (19.2)	158 (21.8)	142 (17.0)			
Seldom (<once/month)	1024 (65.6)	412 (56.7)	612 (73.4)			
Drinking tea beverage						
Always (>5 times/week)	62 (4.0)	43 (5.9)	19 (2.3)	77.87	0.22	<0.001
Usually (3–4 times/week)	125 (8.0)	85 (11.7)	40 (4.8)			
Sometimes (1–2 times/ week)	302 (19.4)	169 (23.3)	133 (15.9)			
Occasionally (1–3 times/month)	377 (24.2)	179 (24.7)	198 (23.7)			
Seldom (<once/month)	694 (44.5)	250 (34.4)	444 (53.2)			
Drinking milk beverage						
Always (>5 times/week)	176 (11.3)	81 (11.2)	95 (11.4)	3.14	0.05	0.535
Usually (3–4 times/week)	187 (12.0)	89 (12.3)	98 (11.8)			
Sometimes (1–2 times/week)	292 (18.7)	147 (20.2)	145 (17.4)			
Occasionally (1–3 times/month)	332 (21.3)	156 (21.5)	176 (21.1)			
Seldom (<once/month)	573 (36.7)	253 (34.8)	320 (38.4)			
Drinking vegetable protein drinks						
Always (>5 times/week)	143 (9.2)	65 (9.0)	78 (9.4)	17.64	0.11	0.001
Usually (3–4 times/week)	170 (10.9)	89 (12.3)	81 (9.7)			
Sometimes (1–2 times/week)	264 (16.9)	149 (20.5)	115 (13.8)			
Occasionally (1–3 times/month)	403 (25.8)	174 (24.0)	229 (27.5)			
Seldom (<once/month)	580 (37.2)	249 (34.3)	331 (39.7)			
